# Branching architecture of tryptophan metabolism determines therapeutic vulnerability in acute myeloid leukemia

**DOI:** 10.1038/s41419-026-09050-z

**Published:** 2026-06-30

**Authors:** Tomasz Pienkowski, Marianna Ciwun, Anna Tankiewicz-Kwedlo, Aleksandra Golonko, Lukasz Bolkun, Jaroslaw Piszcz, Waldemar Priebe, Dariusz Pawlak

**Affiliations:** 1 Laboratory of Metabolomics and Proteomics, Clinical Research Centre, Medical University of Bialystok, Bialystok, Poland; 2 Department of Pharmacodynamics, Medical University of Bialystok, Bialystok, Poland; 3 Department of Hematology, Medical University of Bialystok, Bialystok, Poland; 4 Department of Experimental Therapeutics, The University of Texas MD Anderson Cancer Center, Houston, TX USA

**Keywords:** Cancer metabolism, Acute myeloid leukaemia, Acute myeloid leukaemia, Biomarkers

## Abstract

Acute myeloid leukemia (AML) exhibits metabolic reprogramming that supports immune evasion and treatment resistance. The kynurenine pathway (KP) is a key regulator of tumor–immune interactions, yet its downstream organization and clinical relevance in AML remain unclear. Here, we combined in vitro models with patient serum profiling to determine whether KP branching patterns are associated with treatment response. Extracellular KP metabolites were quantified in AML cell lines (HL-60 and MOLM-14) following induction regimens, and quantified circulating KP metabolites in patient serum samples collected from AML patients before and after induction therapy. Treatment was associated with normalization of tryptophan depletion and kynurenine accumulation in responders, indicating partial restoration of systemic KP homeostasis. Notably, baseline (pre-treatment) samples from patients who were later classified as non-responders exhibited a distinct metabolic phenotype characterized by persistent kynurenine elevation, increased anthranilic and kynurenic acid levels, and enrichment of 3-hydroxykynurenine flux, suggesting preferential engagement of oxidative and immunomodulatory KP branches. Among evaluated metabolic indices, the 3-hydroxykynurenine-to-kynurenine ratio demonstrated the strongest discriminatory capacity for distinguishing response to induction therapy (DA: daunorubicin + cytarabine; DAC: daunorubicin + cytarabine + cladribine), outperforming individual metabolite measurements and highlighting functional pathway flux rather than absolute metabolite abundance as a determinant of clinical outcome.

**Figure:**
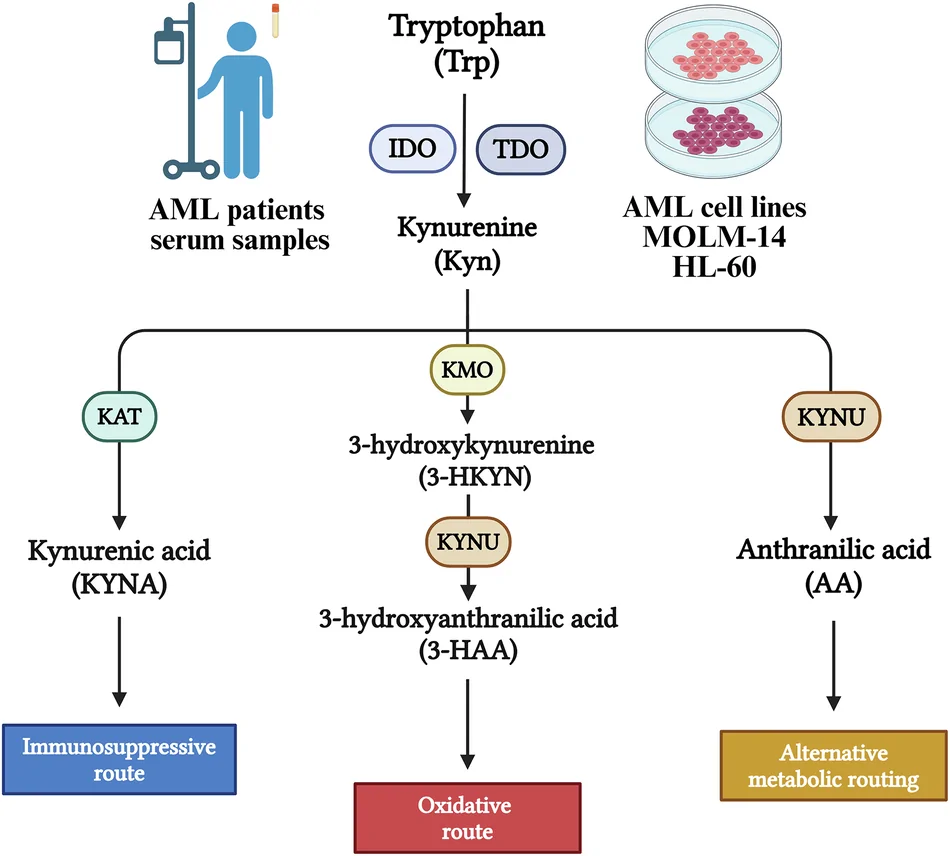

## Introduction

Acute myeloid leukemia (AML) is a heterogeneous hematologic malignancy characterized by clonal expansion of immature myeloid progenitors and leukemic stem cells, resulting in impaired hematopoiesis. The disease arises from accumulated genetic and epigenetic alterations in key regulatory genes, such as *FLT3*, *NPM1*, *DNMT3A*, and *TP53*, which affect cell differentiation and promote proliferation and survival. Among these, *FLT3* internal tandem duplication (FLT3-ITD) is a major adverse driver associated with aggressive disease biology, high relapse rates, and poor outcomes.

Leukemic cells reprogram metabolism to sustain proliferation, adapt to stress, and evade immune surveillance. These metabolic adaptations not only support leukemic cell survival but also alter systemic metabolic homeostasis. Metabolomic studies, including our own previous global liquid chromatography–mass spectrometry (LC–MS)–based study on the same group of patients, showed that AML patients display distinct circulating metabolite profiles compared with healthy individuals, reflecting both tumor-intrinsic metabolism and patient responses [1–5]. These changes are linked to leukemic progression and therapeutic resistance [6].

One metabolic pathway that remains incompletely characterized in AML is the tryptophan–kynurenine pathway (KP), the principal route of tryptophan (Trp) catabolism. Trp is an essential amino acid that supports protein synthesis and immune functions, and its levels are tightly regulated under physiological conditions. The rate-limiting step of KP is catalyzed by indoleamine-2,3-dioxygenase (IDO1/IDO2) or tryptophan-2,3-dioxygenase (TDO), generating kynurenine (Kyn) as the central hub metabolite. Kyn is subsequently metabolized through several enzymatic branches to produce anthranilic acid (AA), 3-hydroxyanthranilic acid (3-HAA), kynurenic acid (KYNA), and 3-Hydroxykynurenine (3-HKYN), each exerting distinct biological activities.

Upregulation of IDO/TDO accelerates Trp depletion and accumulates Kyn and its downstream metabolites [7–9]. This shift has immunological consequences, as reduced Trp availability impairs T-cell proliferation, while Kyn and KYNA activate aryl hydrocarbon receptor (AhR), promoting regulatory T-cell differentiation and immune tolerance [7, 10]. In AML, IDO1 expression has been detected in leukemic blasts and antigen-presenting cells, and elevated circulating Kyn levels correlate with poor prognosis [11]. However, most prior studies have focused on IDO1 expression or the Kyn/Trp ratio alone, without systematically examining downstream KP metabolites or their association with therapeutic outcomes [12–14]. Thus, how differential downstream KP metabolite engagement relates to AML disease biology and treatment response remains a critical knowledge gap.

In this study, we demonstrate that AML is characterized by distinct KP branching signatures that stratify treatment response and reveal differential engagement of oxidative and immunomodulatory metabolic routes. Our findings identify the 3-HKYN/Kyn axis as a novel translational biomarker candidate linking metabolic wiring with clinical therapeutic efficacy.

As an initial exploratory step, we assessed KP metabolites in AML cell line culture media to evaluate tumor-intrinsic Trp catabolism and downstream pathway engagement. These observations guided subsequent clinical analyses quantifying downstream Kyn metabolites. We compared metabolite levels and pathway ratios between patients achieving complete remission and those with refractory disease to determine discriminatory potential. Longitudinal analyses assessed pathway changes before and after therapy in responders. Finally, diagnostic performance was examined using receiver operating characteristic analyses, focusing on Kyn/Trp and 3-HKYN/Kyn ratios.

## Methods

### Study group and ethical approval

AML diagnosis was established according to the World Health Organization (WHO) classification. Leukemic blasts were confirmed by peripheral blood counts and flow cytometry. Cytogenetic and molecular analyses, including fluorescence in situ hybridization for recurrent rearrangements *AML1***::***ETO*, *CBFβ***::***MYH11*, *MLLT3***::***MLL* and mutation screening of *FLT3*, *NPM1*, *CEBPA*, and *TP53*, were performed for risk stratification in accordance with WHO recommendations [15].

Risk stratification followed the European LeukemiaNet recommendations valid at diagnosis (Patients were treated at the Department of Hematology, University Hospital of Bialystok, Poland, between 2019 and 2021). Favorable risk included t(8;21), inv(16), or core binding factor leukemia with CEBPA mutations. Intermediate risk comprised normal karyotype, NPM1 mutations with concurrent FLT3-ITD, and FLT3-ITD without *NPM1* mutation. Adverse risk included del(5q), del(7q), or complex karyotypes defined as three or more cytogenetic abnormalities.

Patients had no evidence of autoimmune, infectious, or metabolic disorders, including dyslipidemia or diabetes. Fasting serum samples were collected in the morning prior to treatment initiation (*n* = 87) and, when applicable, within a defined post-treatment window of 28 ± 7 days from the first dose of chemotherapy in patients who achieved complete remission (*n* = 30) (Table 1).

**Table 1:** Characteristics of the AML patients.

Number of patients	135
Mean (range) age, year	50 (18–67)
Mean (± SD) white blood cell count (G/l)	38.12 (2.0–212.3)
Mean (range) the percentage of the blastic cells in peripheral blood	47 (0–98)
Mean (range) of blast cells percentage in bone marrow	56 (20–93)
**CR after therapy**	**30**
DAC CR	15
DA CR	15
**CR before therapy**	**38**
DAC CR	23
DA CR	15
**NR**	**49**
FLT3-ITD in NR	9
FLT3-WT in NR	40
**Healthy controls**	**18**
Favourable risk in NR FLT3-WT	5
Intermediate risk in NR FLT3-WT	9
Unfavourable risk in NR FLT3-WT	7

Patients were treated using standard 7-day induction chemotherapy regimens in accordance with protocols of the Polish Adult Leukemia Group (PALG). Briefly, cytarabine was administered as a continuous intravenous infusion for seven consecutive days at a dose of 200 mg/m², combined with an anthracycline administered as an intravenous bolus for three consecutive days at a dose of 60 mg/m² in the DA (Daunorubicin + Cytarabine) regimen. Additionally, cladribine was administered as an intravenous bolus for five consecutive days at a dose of 5 mg/m² for the DAC (Daunorubicin + Cytarabine + Cladribine) regimen. Patients were assigned to induction therapy with either DA or DAC according to routine clinical practice at our institution and at the discretion of the treating hematologist, in line with recommendations and evidence from studies conducted by the PALG. The analyzed samples were derived from a biobanked archival cohort [16].

Morphological response was assessed after induction therapy according to the criteria described by Cheson et al. [17]. Complete remission (CR) was achieved in 30 patients following the first induction, whereas 49 patients were classified as non-responders (NR).

The control group comprised 18 age-matched healthy volunteers with a 1:1 male-to-female ratio. Controls were fever-free for at least one week, were not receiving medication, were not pregnant, and had no history of chronic disease.

The Ethics and Research Committee of the Medical University of Bialystok, Poland, granted ethical approval (APK.002.69.2023). The study was conducted in accordance with institutional guidelines at Bialystok University Hospital. Written informed consent was obtained from all participants in accordance with the Declaration of Helsinki.

### Cell cultures

HL-60 (DSMZ ACC-3, lot 30; FLT3-wild-type) and MOLM-14 (DSMZ ACC-777, lot 9; FLT3-ITD) AML cell lines were maintained as suspension monocultures in RPMI-1640 medium supplemented with heat-inactivated fetal bovine serum (10% FBS) and 1% penicillin–streptomycin, at 37 °C in a 5% CO₂ incubator. Cells were expanded to ~90% confluency before therapy.

The standard DA and the DAC treatment regimens were reconstructed using clinical-grade drugs provided by the Department of Hematology. Drugs were applied to each cell line, and after 24h of exposure, cell viability was measured by the CCK-8 assay. Culture media samples were done in 12 replicates, and protein concentration was measured using the Thermo Scientific™ Pierce™ BCA Protein Assay Kit.

To further investigate the relationship between kynurenine pathway modulation and chemotherapy sensitivity, AML cell lines were additionally treated with the IDO1 inhibitor PF-06840003 or exogenous kynurenine Kyn in combination with DA/DAC regimens. Cell viability was assessed after 24, 48, and 72 h using the CCK-8 assay.

### Measurement of kynurenine pathway metabolites

Trp and metabolites of the KP were quantified using high-performance liquid chromatography (HPLC).

Samples were deproteinized by the addition of 25 μL of 2 M perchloric acid to 100 μL of sample, vortexed, incubated at 4 °C for 2 min, and centrifuged at 14,000 rpm for 30 min at 4 °C. Supernatants were used for chromatographic analysis.

Kyn and Trp: Quantified using a Reprospher 100 C18 column (3.5 μm, 2 × 150 mm). Mobile phase: 0.1 M acetic acid and 0.1 M ammonium acetate (pH 4.6) supplemented with 7% acetonitrile, delivered at a flow rate of 0.18 mL/min. Detection: diode-array detector at 365 nm for Kyn and 280 nm for Trp. Column temperature: 24 °C. Injection volume: 2 μL.

3-HKYN: Quantified using HPLC with electrochemical detection (working electrode potential: 0.6 V) on a Waters Spherisorb ODS column (3 μm, 2 × 150 mm) at 24 °C. Mobile phase: 0.1 M triethylamine, 0.1 M phosphoric acid, 0.3 mM EDTA, and 8.2 mM heptane-1-sulfonic acid sodium salt, and 8% acetonitrile; flow rate: 0.22 mL/min; injection 5 μL.

KYNA, AA, and 3-HAA: Measured using a Reprospher 100 C18 column (3.5 μm, 2 × 150 mm) with fluorescence detection (excitation 250 nm, emission 398 nm). Mobile phase: 5 mM zinc acetate and 20 mM acetic acid with 8% acetonitrile; flow rate: 0.18 mL/min; injection 1 μL. Data acquisition and processing: LC ChemStation software. All analyses were conducted at 24 °C.

### Statistical analysis

Statistical analyses and data visualization were performed using GraphPad Prism v10 (GraphPad Software, San Diego, CA, USA). Data distribution was assessed for normality; due to the non-normal distribution of Kyn concentrations, non-parametric tests were applied. Comparisons among more than two groups were conducted using the Kruskal–Wallis one-way analysis of variance, with Dunn’s post hoc multiple comparison test. Pairwise comparisons were performed using the Mann–Whitney U test. Data are presented as median ± standard deviation (SD). All statistical tests were two-tailed, and a *p* value < 0.05 was considered statistically significant.

## Results

### MOLM-14 and HL60 changes in the kynurenine pathway after DA/DAC treatment

To establish whether AML cells differ in intrinsic KP architecture, we first compared basal and treatment-induced KP metabolite profiles in HL-60 and MOLM-14 cells.

Each experimental condition, including controls, was performed in 12 biological replicates per administered therapy. For downstream analyses, cell culture media were collected and subjected to quantitative assessment of KP metabolites. Measurement of metabolites in the culture medium was chosen to capture the net functional output of KP activity, reflected by extracellular Trp depletion and Kyn release, and to ensure mechanistic comparability with serum-based clinical analyses. All metabolite concentrations were normalized to total cellular protein content and expressed per milligram of protein.

**Fig. 1:**
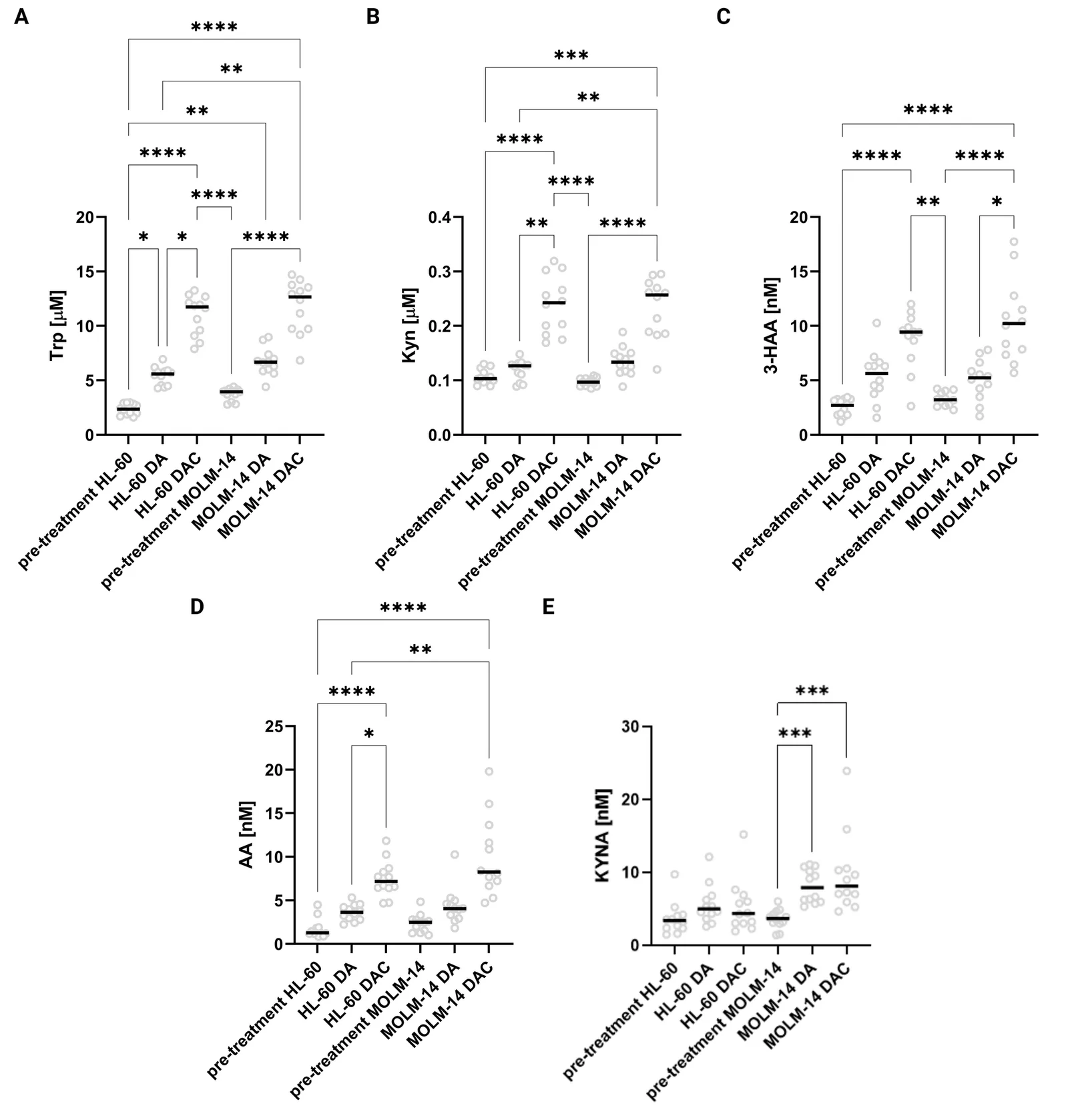
Levels of extracellular kynurenine pathway metabolites in HL-60 and MOLM-14 cell lines, either untreated or treated with DA and DAC therapy. **A** tryptophan (Trp), **B** kynurenine (Kyn), **C** 3-hydroxyanthranilic acid (3-HAA), **D** anthranilic acid (AA), and **E** kynurenic acid (KYNA). *p < 0.05; **p < 0.01; ***p < 0.001; ****p < 0.0001.

Following treatment, Trp concentrations were significantly increased in both cell lines under both therapeutic regimens (Fig. 1A). Kyn levels also increased following treatment; however, statistical significance relative to pre-treatment controls was observed only in the DAC regime (Fig. 1B). Similarly, concentrations of 3-HAA and AA increased under both regimens, with significant differences detected exclusively for DAC-treated cells compared with pre-treatment controls (Fig. 1C, D). KYNA followed the same overall trend, although statistical significance was observed only in MOLM-14 cells following pre- to post-treatment comparison (Fig. 1E).

The activity of KP enzymes was calculated indirectly by the determination of product-to-substrate ratios [18]. The Kyn/Trp ratio, a surrogate marker of TDO/IDO1 activity, was significantly higher in pre-treatment HL-60 media compared with MOLM-14 and with post-treatment conditions (Supplementary Fig. 1A). Following therapy, both cell lines exhibited a significant reduction in the Kyn/Trp ratio irrespective of the regimen. In contrast, the 3-HAA/AA ratio, reflecting anthranilate-3-hydroxylase (A3H) activity, showed no significant changes before or after treatment in either cell line, although pre-treatment HL-60 media displayed a higher median ratio overall (Supplementary Fig. 1B). The AA/Kyn and KYNA/Kyn ratios, indicative of kynureninase (KYNU A) and kynurenine aminotransferase (KAT) activity, respectively, were lower at baseline in HL-60 media and increased after treatment, whereas no significant differences were observed in MOLM-14 media across treatment conditions (Supplementary Fig. 1C, D).

### Does modulation of the kynurenine pathway change chemotherapy sensitivity in AML cell lines?

To assess whether direct modulation of the kynurenine pathway affects AML cell viability during chemotherapy exposure, HL-60 and MOLM-14 cells were treated with exogenous kynurenine (KYN) or the IDO1 inhibitor PF-06840003, either alone or in combination with DA/DAC regimens. Cell viability was measured after 24, 48, and 72 h using the CCK-8 assay (Supplementary Fig. 2).

Addition of PF-06840003 or KYN did not significantly alter cell viability in untreated control conditions or in combination with DA or DAC therapy in either HL-60 (Supplementary Fig. 2A–C) or MOLM-14 cells (Supplementary Fig. 2D–F). Thus, short-term pharmacological modulation of KP activity was not sufficient to induce measurable viability changes in AML monocultures under the tested conditions.

However, clear differences in chemotherapy sensitivity were observed between the two AML cell lines. MOLM-14 cells showed an earlier response to DA and DAC treatment, whereas HL-60 cells displayed a delayed reduction in viability that became more evident after 72 h.

### Serum kynurenine pathway metabolism and enzyme activity before and after DA therapy in responders (CR)

Pre-treatment samples were analyzed separately to capture baseline disease-intrinsic biological and metabolic heterogeneity that may determine therapy susceptibility and predict treatment response independently of therapy-induced changes. Further, DA and DAC regimens should be evaluated as distinct treatment groups because the addition of cladribine in DAC introduces unique mechanisms of action, modifies treatment intensity, and reflects different clinical selection criteria, all of which can drive divergent therapeutic responses and biomarker dynamics that would be obscured if both regimens were analyzed together.

**Fig. 2:**
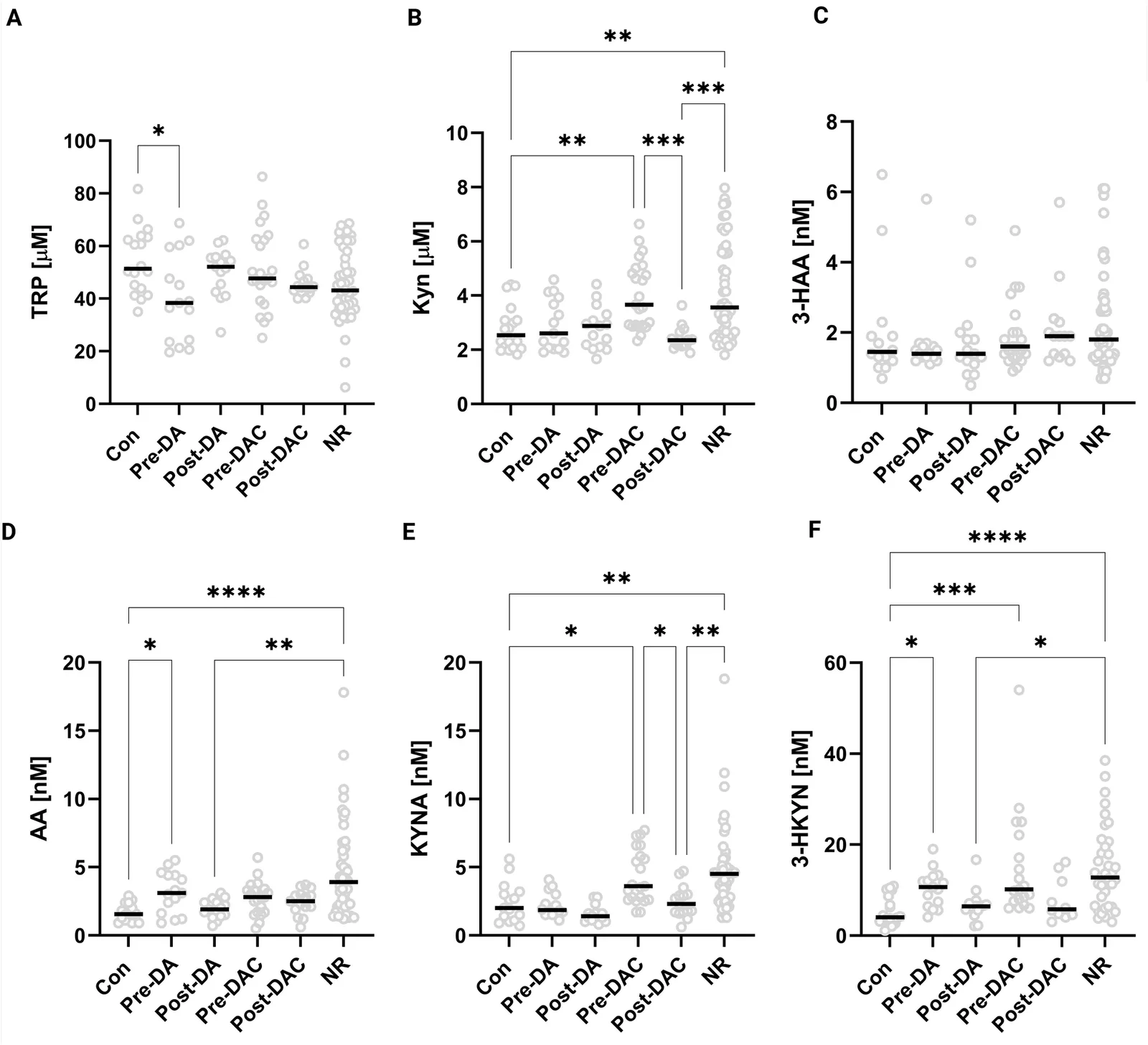
Levels of circulating kynurenine pathway metabolites in controls and AML patients before and after DA or DAC treatment approach. **A** tryptophan (Trp), **B** kynurenine (Kyn), **C** 3-hydroxyanthranilic acid (3-HAA), **D** anthranilic acid (AA), **E** kynurenic acid (KYNA), and **F** 3-Hydroxykynurenine (3-HKYN). *p < 0.05; **p < 0.01; ***p < 0.001; ****p < 0.0001. Data are shown as ranks derived from absolute kynurenine concentrations to reflect non-parametric statistical analysis. Statistical significance was assessed using rank-based tests. Con control, NR non-responders.

Serum Trp concentrations were significantly lower in patients prior to DA therapy compared with controls and increased toward control levels following successful treatment (Fig. 2A). This pattern was not observed in the pre-DAC group; however, despite wide baseline dispersion, post-DAC values converged toward the control median. Kyn concentrations displayed an inverse pattern, with significantly elevated levels in the pre-DAC group that decreased after therapy to values closer to controls (Fig. 2B). NR exhibited persistently higher kynurenine levels compared with both control and post-DAC groups, with medians closer to the pre-DAC cohort. Concentrations of 3-HAA remained stable across all groups (Fig. 2C). In contrast, AA levels were significantly lower in controls than in pre-DA and NR groups, with normalization observed following DA therapy; pre- and post-DAC groups did not differ significantly but showed higher median values overall (Fig. 2D). KYNA followed the opposite trend, with significantly increased levels in pre-DAC and NR patients compared with control and post-DAC (Fig. 2E). Finally, 3-HKYN concentrations were elevated in both pre-treatment groups and in NR patients relative to controls and decreased toward control levels after treatment (Fig. 2F).

**Fig. 3:**
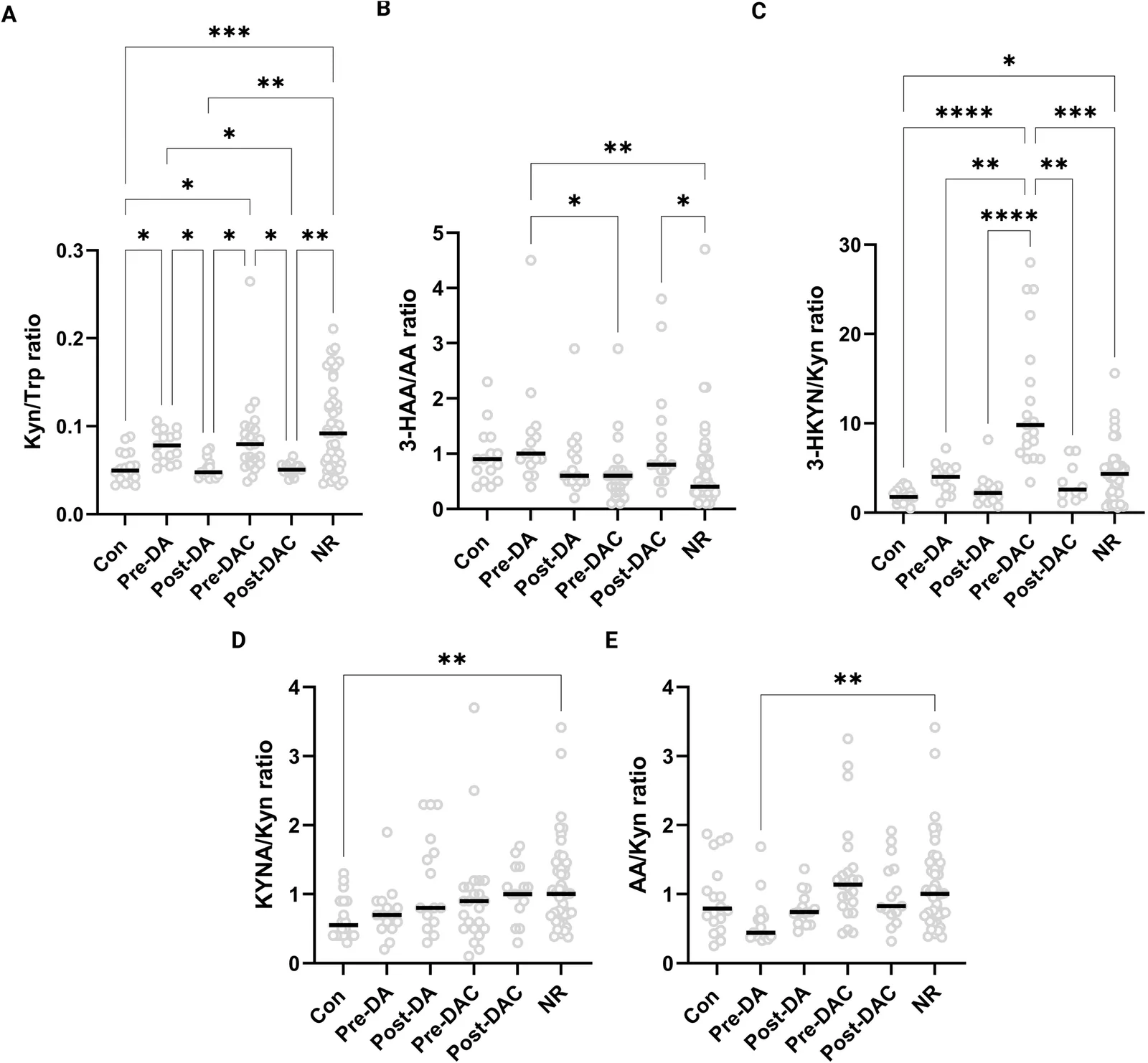
The activity of kynurenine pathway enzymes in controls and AML patients before and after DA or DAC treatment approaches. **A** IDO1, TDO activity (Kyn/Trp ratio), **B** A3H activity (3-HAA/AA ratio), **C** KYNU A activity (AA/Kyn ratio), **D** KAT activity (KYNA/Kyn ratio), and **E** KMO activity (3-HKYN/Kyn). *p < 0.05; **p < 0.01; ***p < 0.001; ****p < 0.0001. Con control, NR non-responders.

The Kyn/Trp ratio was elevated in NR and pre-treatment groups relative to post-treatment samples and decreased toward control levels following therapy (Fig. 3A). The 3-HAA/AA ratio differed significantly between the pre-DA and post-DAC groups and NR; however, median values indicated a decrease after DA and an increase after DAC relative to pre-treatment, changes that did not reach statistical significance (Fig. 3B). Among the ratios examined, the 3-HKYN/Kyn ratio showed the most pronounced discrimination, with higher values in pre-treatment and NR groups compared with control and post-treatment samples (Fig. 3C). The KYNA/Kyn ratio did not differ significantly between pre- and post-treatment conditions, except for a difference between controls and NR, although median values suggested a post-treatment increase (Fig. 3D). The AA/Kyn ratio displayed wide inter-individual variability without statistically significant differences; median values indicated an increase following DA and a decrease following DAC, both converging toward control levels (Fig. 3E).

No statistically significant differences were observed between NR with FLT3-ITD and FLT3 wild-type (Supplementary Figs 3 and 4).

### Analysis of the predictive ability of kynurenine pathway metabolites in patients

**Fig. 4:**
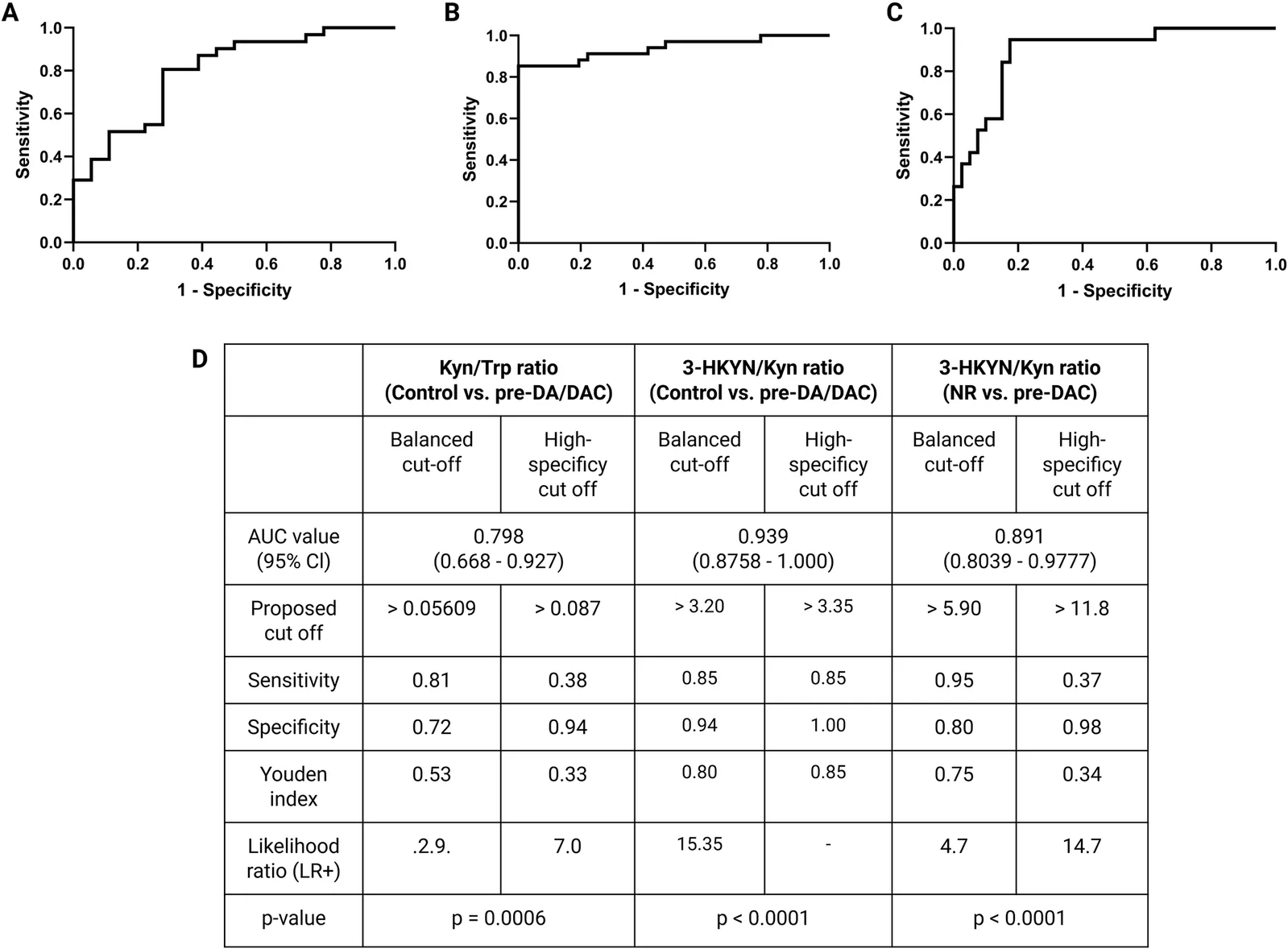
Receiver operating characteristic (ROC) curves predictive ability of the Kyn/Trp ratio. for differentiating between **A** controls from AML patients before therapy that responded to administered treatment based on Kyn/Trp ratio, **B** controls from AML patients before therapy that responded to administered treatment 3-HKYN/Kyn, **C** NR and CR that responded to DAC regime, and **D** Diagnostic performance of the chosen biomarkers based on ROC analysis. CR complete remission, NR non-responders.

ROC analysis assessed the discriminatory performance of the Kyn/Trp and 3-HKYN/Kyn ratios for predicting AML and distinguishing NR from CR before therapy (Fig. 4A, B, C). Responders to DA/DAC were analyzed collectively; both ratios showed clear separation from controls. To reflect different clinical scenarios, both a Youden’s index-based optimal cut-off and a high-specificity cut-off were reported (Panel D). Additionally, the 3-HKYN/Kyn ratio discriminated NR from pre-treatment DAC samples of patients who subsequently achieved CR. No other metabolites or their ratios demonstrated discriminatory performance in any comparisons.

## Discussion

### Differential KP architecture in AML cell lines and remodeling under DA and DAC therapy

At baseline, HL-60 showed a significantly higher Kyn/Trp ratio than MOLM-14, implying greater intrinsic KP-engagement. This distinction aligns with AML literature reporting constitutive and inducible IDO1 activity in leukemic cells, where interferon-γ and inflammatory cues can amplify Trp catabolism and immune escape; such inducibility is observed in AML cells and is a recurring theme in AML-focused systematic synthesis [19].

Following treatment, both cell lines showed increased extracellular Trp and increased Kyn but reduced Kyn/Trp ratio. We speculate that therapy reduces net Trp-Kyn conversion relative to Trp availability, even while absolute Kyn in the medium can rise through release from stressed/dying cells or reduced downstream consumption. This apparent paradox is widely encountered when activity is inferred from extracellular steady-state pools rather than direct enzymatic flux, and it underscores that Kyn/Trp is best treated as a functional surrogate of pathway engagement rather than a direct measurement of enzymatic velocity [20].

A second, more mechanistically discriminating signal emerges from the downstream ratios. In HL‑60, AA/Kyn and KYNA/Kyn ratios increased after therapy, whereas MOLM‑14 displayed relative stability across treatment conditions. This suggests that HL‑60 undergoes treatment-induced redistribution of Kyn metabolism toward KYNU-driven AA formation and KAT-driven KYNA formation, while MOLM‑14 maintains a comparatively fixed branch allocation. Such “branch rebalancing” is supported by cancer-wide datasets showing that KYNU upregulation is linked to immunosuppressive microenvironmental programs and poor prognosis across multiple tumor types, including AML transcriptomic analyses, implying that KYNU can be integrated into tumor-adaptive stress programs [21].

Notably, 3‑HAA and AA increased under both regimens, with stronger statistical support under DAC. This pattern is consistent with a model in which epigenetic therapy can reshape immune-metabolic gene expression and stress responses, while clinical literature also emphasizes that hypomethylating agents can interact with immunosuppressive circuits, including IDO-associated programs [22]. Importantly, the direction of change in HL‑60 ratios (increasing AA/Kyn and KYNA/Kyn) predicts that, even if “front-end” IDO/TDO engagement falls, the remaining Kyn pool may be more actively partitioned into immunomodulatory ligands (KYNA) and AA-related side products, each of which can shape immune phenotypes via AHR and related pathways [23].

A summary of this section is presented in S. Table 1.

### Limited effects of short-term KP modulation on AML monoculture viability

Additionally, in vitro experiments demonstrated that short-term pharmacological modulation of KP activity using the IDO1 inhibitor PF-06840003 or exogenous Kyn did not significantly alter AML cell viability under monoculture conditions. Importantly, this observation should not be interpreted as evidence against KP involvement in AML biology. Rather, it suggests that KP activity may not directly determine short-term leukemic cell survival in simplified in vitro systems lacking immune and stromal components.

This distinction is biologically relevant, as the immunomodulatory functions of KP are strongly linked to inflammatory signaling, cytokine exposure, and microenvironmental interactions that are absent in monoculture conditions. Indeed, IDO1 activity is highly inducible by inflammatory mediators such as IFN-γ, and the functional consequences of KP activation frequently emerge through effects on immune regulation rather than direct cytotoxic dependency of tumor cells [24, 25]

Notably, despite the limited impact of direct KP modulation itself, MOLM-14 and HL-60 cells displayed distinct kinetics of response to DA/DAC treatment, with MOLM-14 showing earlier therapy sensitivity and HL-60 demonstrating delayed viability reduction. These observations support the concept that the KP organization may reflect broader treatment-adaptive and immunometabolic states rather than direct control of basal AML cell viability.

### Clinical serum metabolite profiles and prognostic ratios align with KMO-biased disease states

Clinical serum analyses recapitulated the AML cell line findings (Supplementary Table 2). Prior to therapy, AML patients (DA cohort) exhibited reduced Trp and elevated Kyn, with partial normalization in treatment responders and persistence of elevated indices in NR. AML-specific clinical studies demonstrate that elevated serum Kyn and/or elevated Kyn/Trp correlate with poor survival, including classic evidence linking higher Kyn/Trp to shorter survival in AML [26]. A broader AML literature base also supports that IDO1 expression in leukemic cells is associated with unfavorable prognosis [27]. In addition, AML transcriptomic work indicates that IDO1-centered immune gene signatures can predict survival and act as adverse prognostic markers, supporting the view that KP engagement is linked to disease-defining immune–metabolic programs [28].

Importantly, ROC analyses identified 3-HKYN/Kyn as a stronger discriminator of refractory disease than Kyn/Trp. KMO is widely described as a critical branchpoint controlling entry into a metabolically “toxic” arm, with 3-HKYN, a metabolite that drives oxidative injury and apoptotic pathways. This makes 3-HKYN/Kyn mechanistically more specific than Kyn/Trp, which is sensitive to systemic inflammation, nutrition, hepatic metabolism, and renal clearance [29]. Although AML-specific large-scale validations of 3-HKYN/Kyn are still limited compared with Kyn/Trp, the broader oncology literature supports the principle that downstream toxic-arm metabolites can carry independent prognostic information (e.g., circulating 3-HKYN and QA) [20]. Importantly, finding that 3-HKYN/Kyn also discriminates NR patients from pre-treatment DAC responders suggests a branch allocation phenotype that may reflect intrinsic resistance biology (e.g., redox adaptation) rather than simply tumor burden.

Integrating cell line and serum data, a coherent model emerges in which AML simultaneously promotes upstream Trp depletion and Kyn accumulation while favoring downstream KMO-skewed that enriches routing to oxidative/toxic intermediates.

Biologically, 3-HKYN is described as a free-radical donor and oxidative stressor, and KMO overexpression or increased flux is linked to oxidative stress marker induction in cellular systems [28, 30]. More recently, experimental evidence in bone and stromal contexts indicates that exposure to 3-HKYN and 3-HAA can induce ROS-linked apoptosis and impair osteogenic differentiation, supporting the biological plausibility that these metabolites can remodel the marrow niche in AML-relevant ways [31].

Immunologically, KP metabolites operate at multiple regulatory levels: Trp depletion suppresses effector T cell proliferation, while downstream metabolites directly modulate antigen-presenting cell behavior and T cell responses. For example, 3-HAA can induce tolerogenic features such as HLA-G expression in dendritic cells and reduce T cell stimulatory capacity, supporting a mechanism by which KYNU/A3H-side routing may consolidate tolerance [32]. Experimental immunology also demonstrates that 3-HAA can inhibit CD8 + T cell proliferation under cytokine-driven conditions, consistent with direct suppression of cytotoxic effector programs [33]. These observations connect directly to serum results in which disease states show elevation of KMO- and KYNU-linked metabolites alongside elevated Kyn indices, creating a metabolite landscape compatible with both effector suppression and tolerogenic antigen presentation.

Recent work demonstrated that Kyn secreted by AML cells can signal to osteoblasts via serotonin receptor pathways to promote leukemia-supporting programs, highlighting that Kyn is not only a tolerance marker but a marrow-active intercellular signaling metabolite [34]. This provides a plausible bridge between systemic serum markers and local bone marrow remodeling, suggesting that elevated Kyn and KMO-arm products may support both immune evasion and niche conditioning. Moreover, KMO expression itself has been repeatedly linked to tumor aggressiveness and prognosis in non-AML malignancies (e.g., hepatocellular carcinoma), positioning KMO as a tumor-relevant enzyme rather than a purely neurologic/immunologic target [35].

### Therapeutic and translational implications

Our findings suggest that therapeutic strategies targeting KP in AML should move beyond single-node strategies (IDO1-only) toward branch-aware multi-node modulation. Contemporary oncology reviews caution explicitly that focusing solely on IDO1 ignores the immune-suppressive potential of downstream KP enzymes and metabolites, advocating broader pathway reasoning [22, 36].

A rational therapeutic concept is KMO inhibition to reduce 3-HKYN production (and downstream oxidative/toxic pressure), paired with strategies that prevent compensatory diversion into alternative immunosuppressive ligands. KMO inhibition is mechanistically grounded by data showing KMO’s branchpoint role and 3-HKYN’s oxidative injury potential; KMO inhibition reduces oxidative stress-associated pathology in non-cancer severe inflammatory states, providing proof-of-principle that systemic KMO modulation is feasible and biologically impactful [29]. However, KMO blockade predictably elevates upstream Kyn availability, which can increase AHR-ligand signaling unless counterbalanced; therefore, pairing KMO inhibition with AHR pathway modulation or upstream Kyn-limiting strategies could be necessary to avoid shifting suppression from “toxic arm” to “ligand arm” [36].

Targeting KYNU is conceptually attractive because KYNU upregulation correlates with immunosuppressive microenvironmental signatures and poorer prognosis across cancers, and KYNU-focused work suggests it could be a therapeutic node for reshaping immune infiltration states [37]. Yet KYNU inhibition also raises branch logic concerns, as blocking AA production may leave more Kyn available for KMO or KAT, potentially increasing 3-HKYN or KYNA unless KMO is concurrently controlled. This branch compensation can be observed in our HL-60 post-therapy ratio shifts, where therapy rebalances branches rather than uniformly silencing the pathway.

Regarding our ROC findings on Kyn/Trp and 3‑HKYN/Kyn as actionable biomarkers, AML clinical literature already supports Kyn/Trp as a survival-linked indicator and Kyn itself as prognostic. Our data extends this framework by indicating that 3‑HKYN/Kyn can better discriminate response vs NR, consistent with the general oncology principle that downstream toxic-arm metabolites can add prognostic information beyond Kyn/Trp [26]. Notably, similar ratio concepts have appeared in other hematologic malignancies, suggesting wider clinical plausibility for “KMO vs KAT balance” indices [38].

## Conclusions

Collectively, our cell-line and serum findings support a branch-aware model of KP dysregulation in AML. Upstream Trp catabolism (captured by Kyn/Trp) reflects systemic immune–metabolic activation and tolerance, whereas downstream pathway routing at the level of KMO (captured by 3-HKYN/Kyn) marks a shift toward the oxidative toxic branch that is strongly associated with NR and may define a therapeutically relevant phenotype. These findings identify KP branch allocation as both a mechanistic feature of AML biology and a potential translational biomarker axis warranting further validation.

### Limitations

The metabolite ratios applied in this study should be interpreted as proxy indicators of relative pathway routing rather than direct measurements of enzymatic activity. Although indices such as KYN/TRP or 3-HAA/AA are commonly used to infer differential engagement of IDO1-, KMO-, KYNU-, or KAT-dependent branches, they do not quantify enzyme expression or catalytic function directly. Accordingly, the present findings describe patterns of pathway organization and metabolite distribution, without establishing enzyme-level regulation.

In addition, the cellular origin of circulating kynurenine metabolites cannot be resolved. The observed signals may reflect contributions from leukemic blasts, immune cells within the microenvironment, or systemic host responses to chemotherapy. Thus, the results represent integrated, system-level metabolic signatures rather than compartment-specific mechanisms.

The in vitro experiments were performed under monoculture conditions lacking immune and stromal components of the AML microenvironment, which may limit the interpretation of KP modulation effects observed in the cell-line models.

Finally, the predictive analyses presented in this study were performed within a single cohort and were not externally validated. Therefore, the identified KP-related ratios should be interpreted as preliminary observations requiring further investigation in independent AML cohorts.

## Supplementary information

Supplemental material

S.Figure 1

S.Figure 2

S.Figure 3

S.Figure 4

S.Table 1

S.table 2

## Data Availability

Data supporting the findings of this study are available from the corresponding author upon reasonable request. Interested researchers should contact the corresponding author for access to the data.
