# Supplementary material for: Branching architecture of tryptophan metabolism determines therapeutic vulnerability in acute myeloid leukemia
Source: Cell Death Dis. 2026 Jun 30;17(1):812. doi: 10.1038/s41419-026-09050-z (PMC13586247; doi:10.1038/s41419-026-09050-z)
Supplement: Supplementary file 1 [file 41419_2026_9050_MOESM2_ESM.pdf]

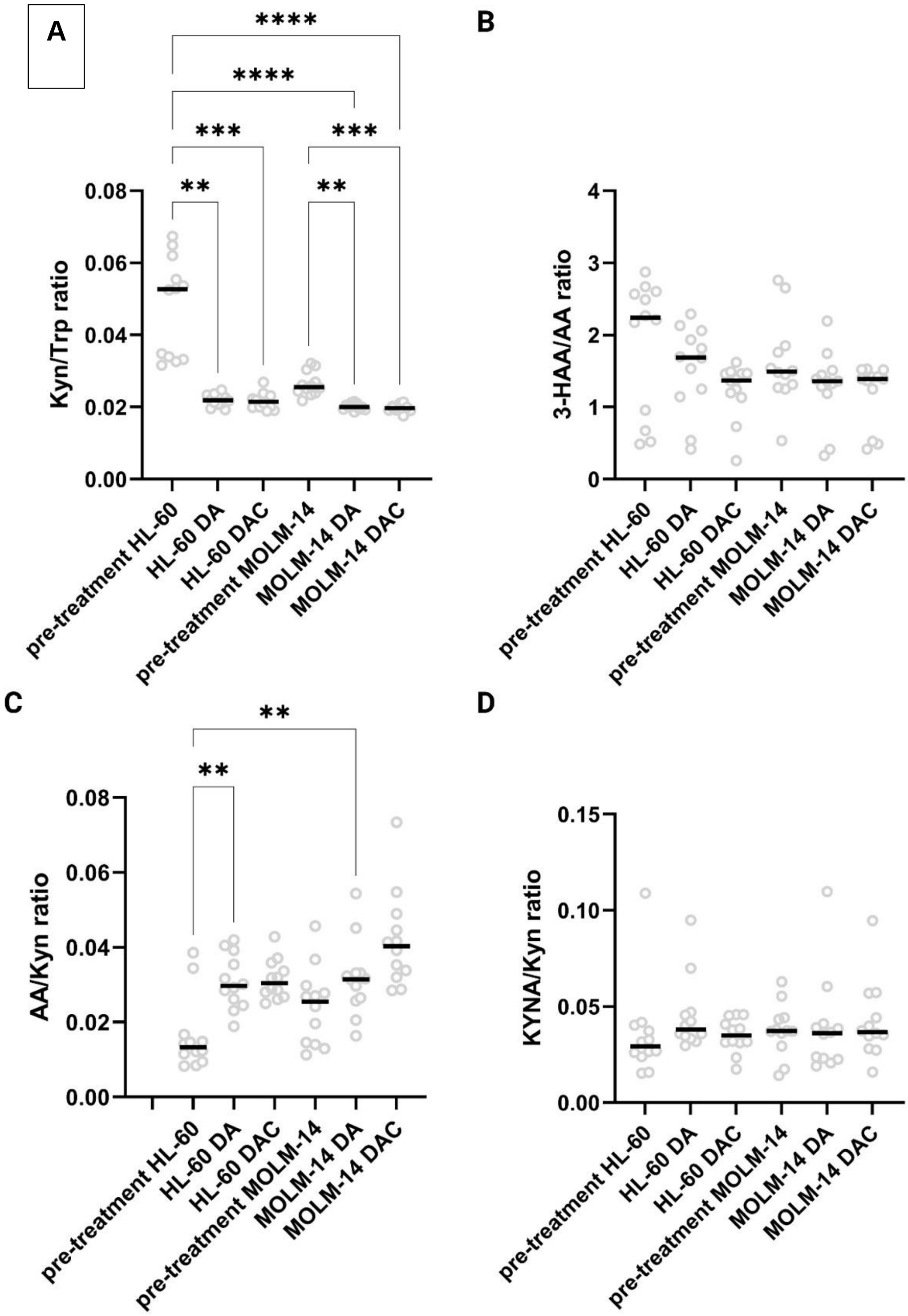
A
B
C
D
Kyn/Trp ratio
3-HAA/AA ratio
AA/Kyn ratio
KYNA/Kyn ratio
**
***
****
pre-treatment HL-60
HL-60 DA
HL-60 DAC
pre-treatment MOLM-14
MOLM-14 DA
MOLM-14 DAC
