# Supplementary material for: Branching architecture of tryptophan metabolism determines therapeutic vulnerability in acute myeloid leukemia
Source: Cell Death Dis. 2026 Jun 30;17(1):812. doi: 10.1038/s41419-026-09050-z (PMC13586247; doi:10.1038/s41419-026-09050-z)
Supplement: Supplementary file 2 [file 41419_2026_9050_MOESM3_ESM.pdf]

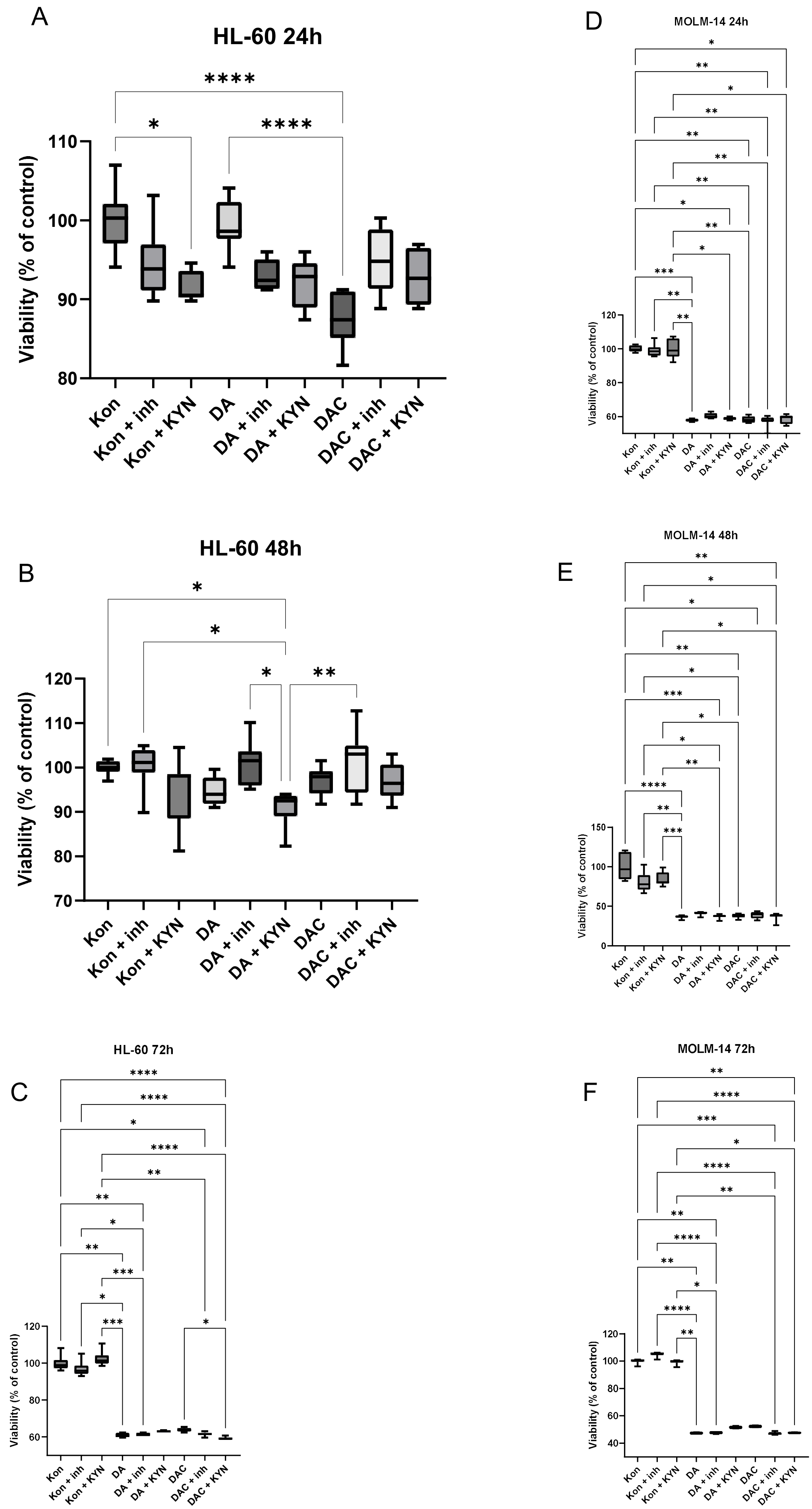
A
HL-60 24h
Viability (% of control)
110
100
90
80
Kon
Kon + inh
Kon + KYN
DA
DA + inh
DA + KYN
DAC
DAC + inh
DAC + KYN
B
HL-60 48h
120
110
100
90
80
70
C
HL-60 72h
120
100
80
60
D
MOLM-14 24h
120
100
80
60
E
MOLM-14 48h
150
100
50
0
F
MOLM-14 72h
120
100
80
60
40
