# Supplementary material for: Branching architecture of tryptophan metabolism determines therapeutic vulnerability in acute myeloid leukemia
Source: Cell Death Dis. 2026 Jun 30;17(1):812. doi: 10.1038/s41419-026-09050-z (PMC13586247; doi:10.1038/s41419-026-09050-z)
Supplement: Supplementary file 3 [file 41419_2026_9050_MOESM4_ESM.pdf]

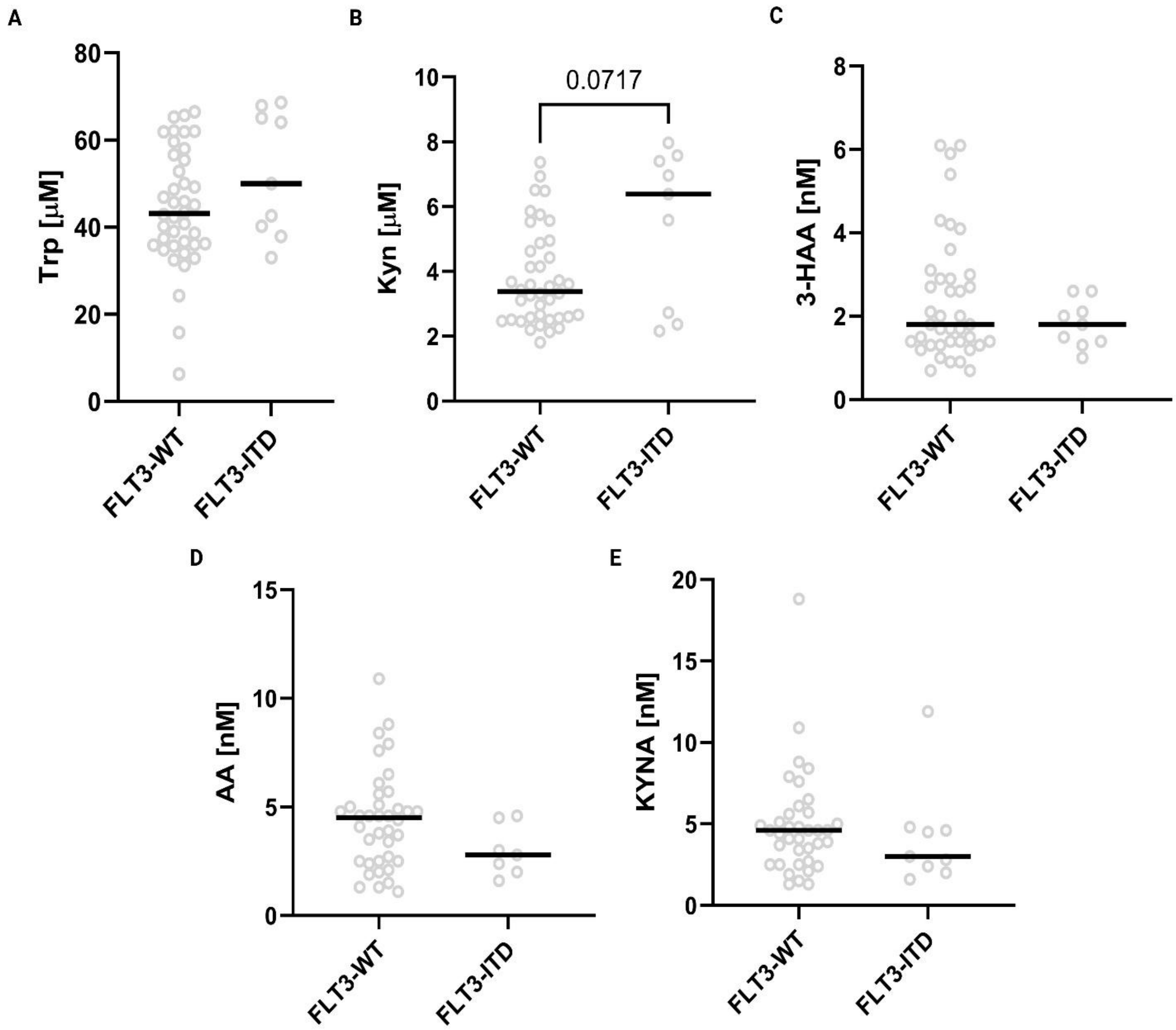
A
B
C
D
E
Trp [μM]
Kyn [μM]
3-HAA [nM]
AA [nM]
KYNA [nM]
0.0717
FLT3-WT
FLT3-ITD
80
60
40
20
0
10
8
6
4
2
0
15
10
5
0
20
15
10
5
0
