# Supplementary material for: Branching architecture of tryptophan metabolism determines therapeutic vulnerability in acute myeloid leukemia
Source: Cell Death Dis. 2026 Jun 30;17(1):812. doi: 10.1038/s41419-026-09050-z (PMC13586247; doi:10.1038/s41419-026-09050-z)
Supplement: Supplementary file 4 [file 41419_2026_9050_MOESM5_ESM.pdf]

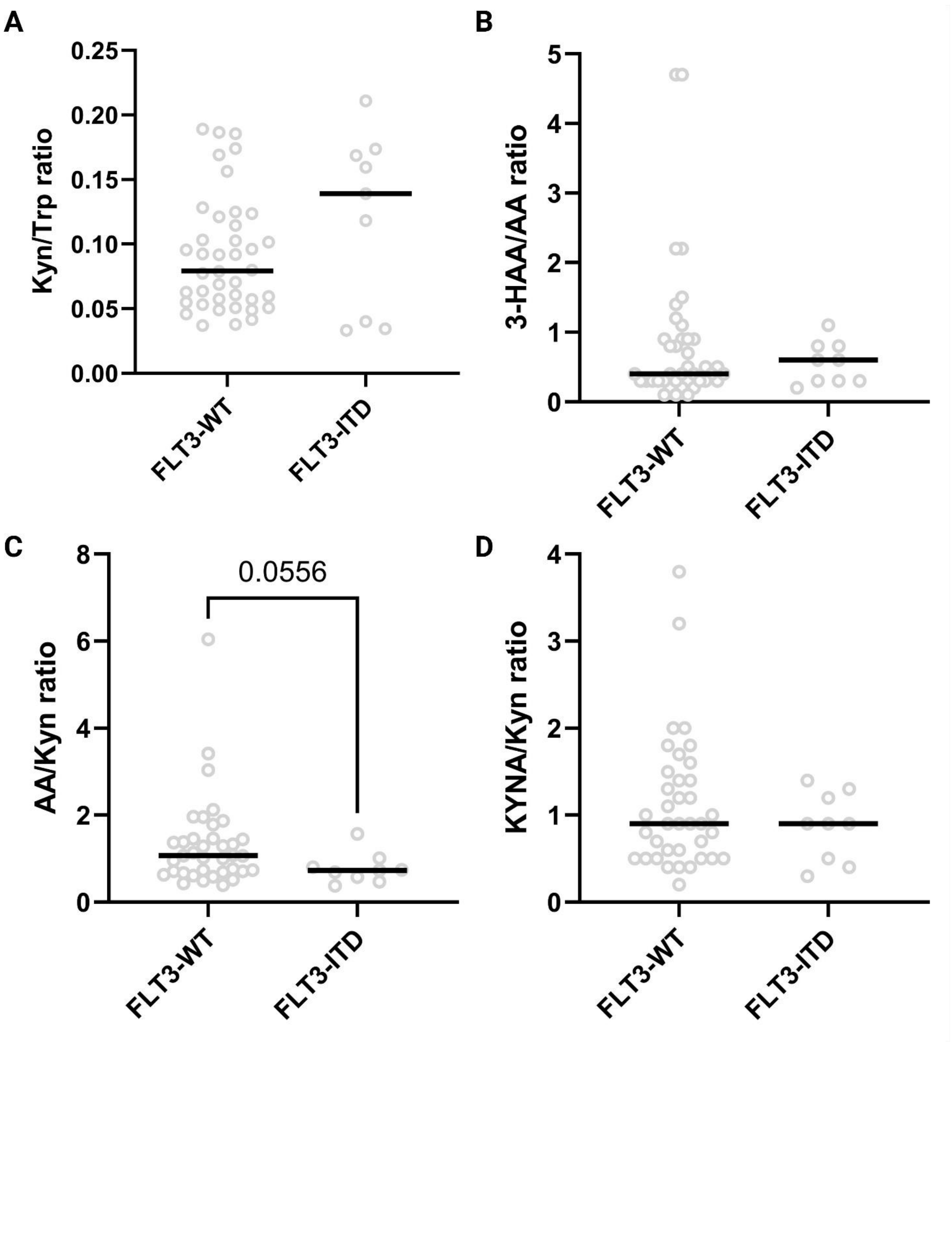

A
0.25
0.20
0.15
0.10
0.05
0.00
Kyn/Trp ratio
FLT3-WT
FLT3-ITD
B
5
4
3
2
1
0
3-HAA/AA ratio
FLT3-WT
FLT3-ITD
C
8
6
4
2
0
0.0556
AA/Kyn ratio
FLT3-WT
FLT3-ITD
D
4
3
2
1
0
KYNA/Kyn ratio
FLT3-WT
FLT3-ITD
