# Supplementary material for: Branching architecture of tryptophan metabolism determines therapeutic vulnerability in acute myeloid leukemia
Source: Cell Death Dis. 2026 Jun 30;17(1):812. doi: 10.1038/s41419-026-09050-z (PMC13586247; doi:10.1038/s41419-026-09050-z)
Supplement: Supplementary file 5 [file 41419_2026_9050_MOESM1_ESM.docx]

**Supplementary figures and tables description**

**S.Figure 1. The activity of kynurenine pathway enzymes between untreated and treated with DA or DAC therapeutic approaches in HL-60 and MOLM-14 cell line culture media.** The activity of individual KP enzymes were calculated indirectly, by the determination of product/substrate ratios, (A) IDO1, TDO activity (Kyn/Trp ratio), (B) A3H activity (3-HAA/AA ratio), (C) KYNU A activity (AA/Kyn ratio), (D) KAT activity (KYNA/Kyn ratio). *p < 0.05; **p < 0.01; ***p < 0.001; ****p < 0.0001.

**S.Figure 2.** **Effects of DA and DAC treatment combined with IDO1 inhibition or exogenous kynurenine supplementation on AML cell viability assessed using the CCK-8 assay.** (A–C) HL-60 cells after 24 h (A), 48 h (B), and 72 h (C) of incubation. (D–F) MOLM-14 cells after 24 h (D), 48 h (E), and 72 h (F) of incubation. Cell viability was evaluated following treatment with DA or DAC in the presence or absence of the IDO1 inhibitor PF-06840003 or exogenous L-kynurenine (KYN).

**S.Figure 3. The kynurenine pathway metabolites in No Response (NR) patients with FLT3-ITD mutation and FLT3-WT.** (A) kynurenine (KYN), (B) tryptophan (TRP), (C) 3-hydroxyanthranilic acid (3-HAA), (D) anthranilic acid (AA), and (E) kynurenic acid (KYNA).

**S.Figure 4. The kynurenine pathway metabolites in No Response (NR) patients with FLT3-ITD mutation and FLT3-WT.** (A) IDO1, TDO activity (KYN/TRP ratio), (B) KMO activity (3-HKYN/KYN ratio), (C) KYNU A activity (AA/KYN ratio), and (D) KAT activity (KYNA/KYN ratio).

**S.Table 1.** Summary table of in vitro remodeling signals

**S.Table 2.** Summary table of clinical directionality and outcome association.
